# Feelings of Blameworthiness and Their Associations With the Grieving Process in Suicide Mourning

**DOI:** 10.3389/fpsyg.2020.00610

**Published:** 2020-04-21

**Authors:** William Feigelman, Julie Cerel

**Affiliations:** ^1^Sociology Department, Nassau Community College, Garden City, NY, United States; ^2^College of Social Work, University of Kentucky, Lexington, KY, United States

**Keywords:** suicide bereavement, guilt feelings, blameworthiness, complex grief, grief difficulties

## Abstract

**Aims:**

This study focuses on identifying the correlates associated with the emergence of feelings of blameworthiness associated with a suicide or other traumatic death and its associations with grief complications.

**Methods:**

Based on a mailed questionnaire survey of 575 mostly white and economically advantaged bereaved parents, 462 who lost a child to suicide, 48 to a drug overdose, 37 to ordinary accidents, and 24 to natural causes, we utilized chi-square tests, correlations and multiple regression analysis to compare and contrast patterns in the data.

**Results:**

Findings showed feelings of blameworthiness associated with grief difficulties, complicated grief, PTSD, depression and other mental health difficulties among suicide bereaved parents. Results among suicide bereaved parents also showed that being stigmatized by socially significant others, having a mixed or negative relationship with the deceased child prior to the death and a less happy marriage, among those presently married couples, all contributed to higher feelings of blameworthiness among these bereaved.

**Conclusion:**

Based on these findings, feelings of blameworthiness could serve as a good shorthand indicator of grief problems since it correlates so well with other grief difficulties and mental health problem measures. The importance of peer support is essential for avoiding the downward spiral associated with feelings of blameworthiness that can occur at any time during the grieving process.

## Introduction

It is almost axiomatic in the bereavement literature that the suicide bereaved will experience feelings of guilt and blameworthiness following a loved one’s death. Typical of such viewpoints is a statement made by the Mayo Clinic staff which appears in one of their on-line grief support pamphlets aimed at helping the suicide bereaved.

“When a loved one dies by suicide, overwhelming emotions can leave you reeling. Your grief might be heart wrenching. At the same time, you might be consumed by guilt – wondering if you could have done something to prevent your loved one’s death.” (Retrieved from mayoclinc.org website, 9/23/19, healthy-lifestyle/end of life/in-depth/suicide).(Mayo Clinic On-line Grief Pamphlet, 2019)

These expectations of feeling guilty and blameworthy in the death are so widely shared that there are few researches probing into whether there are variations in these feelings from one suicide bereaved person to another and what might explain why some of those bereaved will experience greater feelings of blameworthiness than others (Cerel et al., 2008; Hanschmidt et al., 2016; Shields et al., 2017; Sheehan et al., 2018).

Yet, it is widely known and accepted among clinicians and researchers alike that the suicide bereaved will usually be haunted by feelings of guilt, self-doubt about what they could or should have done prior to the death to avoid it, anger at the deceased and shame about the death itself that gives suicide its uniquely highly distressing elements for the bereaved, leading them to be prone to more complicated grief (Jordan, 2001; Cerel et al., 2008; Dyregrov et al., 2012). Yet, despite this widespread understanding of the importance of self-blaming and guilt in the grieving process few studies have focused precisely upon assessing the impact of blameworthiness itself upon a suicide mourners’ grief.

In this report, using existing survey data from bereaved parents we examine the variations in feelings of blameworthiness as they occur at the time of the death and as they may change at the time of the research interview; we also explore which social demographic and experiential factors are associated with greater feelings of blameworthiness, among some bereaved compared to others. We also explore the variations in experiencing grief problems, such as grief difficulties, complicated grief, PTSD, psychological problems, depression and post-traumatic growth among those experiencing greater or lesser degrees of feelings of blameworthiness.

We hypothesized that suicide and drug death bereaved parents would experience greater feelings of blameworthiness compared to other bereaved parents whose children died from natural causes and or from ordinary accidents, owing to the social disapproval associated with the former types of deaths. In our previous work we found great similarities in exposures to stigmatization and grief difficulties among suicide and drug death bereaved parents, compared to other accident and natural death bereaved parents (Feigelman et al., 2011). We also anticipated that when parents had closer and more positive relationships with their children prior to the death their feelings of blameworthiness would be lessened compared to when parents had mixed or conflicted relationships with their children. We expected the latter group would feel more blameworthy since they stand in a normatively anomalous position by having negative feelings toward their child prior to the death. We also expected that feeling stigmatized by the death, more common in suicide and drug death cases, would increase feelings of blameworthiness, as these bereaved would feel less supported and less validated. In addition, we also anticipated that more grief problems, complicated grief, PTSD, psychological problems and depression would be highly associated with feelings of blameworthiness, and that this would be associated inversely with time after the loss and differences in post-traumatic or personal growth. We anticipated that a statistical testing of all these associations would support these hypotheses and yield deepened understandings on how feelings of blameworthiness emerge and their impact upon the grieving process.

## Materials and Methods

### Participants and Procedures

This report is based upon a convenience sample of 575 bereaved parents drawn primarily from the ranks of peer support group affiliates from the American Association of Suicidology, the American Foundation of Suicide Prevention, the Compassionate Friends and several other smaller groups of bereaved parents, such as the Parents of Suicide Support Group, the Angels of Addiction members, Grief Recovery After Substance Use Passing affiliates, popularly known as GRASP Groups. We also asked several therapists to refer former and current bereaved parent patients to complete our surveys. All survey respondents were also requested to refer additional potential respondents, as well. Most of the surveys were collected from a mailed survey instrument during 2006 and 2007, although in 2009, an additional wave of 37 surveys were collected from drug death bereaved parents. The overall response rate was 72%. While a complete methodological procedures statement will be found in Feigelman et al. (2012, pp. 22–25) potential respondents were sent letters requesting their cooperation. Once they returned letters of interest, they were mailed surveys, consent forms and post-paid return envelopes. The survey form was 27 pages long. Surveys included a most comprehensive array of questions on their socio-demographic characteristics, detailed questions pertaining to their children’s deaths, such whether they had found their child’s body, their relationships to their child at the time of the death; time since the loss, etc. A wide variety of questions were asked pertaining to grief experiences, grief problems, complicated grief, mental health, psychological problems, depression, PTSD and post-traumatic growth.

Surveyed bereaved parents experienced the death of a child anywhere from less than a month after the death until as long as 40 years afterward. All respondents in this survey fit into one of four types of death losses: suicide, drug overdose, ordinary accident or natural death circumstances.

### Measurements

Two questions about feelings of blameworthiness were included in the survey that were never analyzed previously: (1) “People losing loved ones sometimes feel blameworthy that things they may have done (or have not done) could have contributed to the person’s death. Does this apply to you and how you felt during the first weeks after your child’s death.” (2) “Presently, do you feel at all blameworthy in the death.” Respondents were asked to express their agreement to both statements on a five-point scale from (a) Almost completely to (e) Almost not at all.

Theoretically it appeared that lingering feelings of blameworthiness would be the most germane item for us to focus on in this investigation. We therefore directed our attention primarily on whether the respondent felt blameworthy on a five-point scale at the present time. 570 respondents answered this question, yielding a mean of 3.6, SD = 1.22, with a range of 1–5 with lower numbered responses exhibiting greater feelings of self-blame.

To measure grief difficulties, we used an abbreviated version of The Grief Experience Questionnaire (GEQ; Barrett and Scott, 1989). The original GEQ scale consisted of 55 items. Following the lead of Bailley et al. (2000) who performed a factor analysis of the scale and identified eight distinct factors within it, we selected the two highest factor-loaded items for each of the eight factors to form our 16-item abbreviated scale. Our abbreviated scale yielded an internal consistency (Cronbach’s alpha) coefficient of 0.87. The brief GEQ scale was answered by 522 respondents, yielding a mean of 39.1 (SD = 11.5) with scores that ranged from a low of 16 to a high of 80.

We employed a Complicated Grief scale (Prigerson, 2002); this scale was answered by 541 respondents, yielding a mean of 27.9 (SD = 8.9), with scores ranging from 11 to 51. The 13 item scale yielded an alpha coefficient of 0.89. We also utilized the Impact of Events Scale (Horowitz et al., 1979) to measure PTSD. The Impact of Events Scales, was completed by 522 respondents showing a mean score of 33.3 (SD = 8.9) and a range of 14 to 56. The 14-item IES scale yielded an alpha coefficient of 0.89.

We utilized an eight-item measure of depression (Wethington et al., 1998). It was based on responses to an initial screening question: “During the past year, was there ever a time when you felt sad, blue, or depressed for 2 weeks or more in a row?” Those answering affirmatively were asked seven additional questions querying about loss of interest, energy, appetite, concentration, feelings of worthlessness, morbid ideation and sleep disturbance. An 8-point scale (no = 0; yes = 1) was created for responses to these eight questions. In our sample, the alpha coefficient for the scale was 0.92. The depression scale was completed by 511 respondents with a mean of 4.34 (SD = 3.1), with scores ranging from 0 to 8.

We also created an index of personal psychological problems by combining several questions that had been asked in the Midlife Development Survey. The survey asked respondents to self-rate their mental or emotional health: “How about your mental or emotional health? Is it poor, fair, good, very good, or excellent?” Then, we also counted the previously mentioned depression screening question. Survey respondents were also asked to count the number of days in the past 30-day period when they were unable to go to work or had to cut back normal household activities because of mental health difficulties. In addition, they were also asked a life satisfaction question: “At present, how satisfied are you with your life – a lot, somewhat, a little, or not at all/none at all?” These responses were associated with one another with correlation coefficients ranging from 0.20 to 0.52. Summing together responses of (a) poor or fair mental health reports, (b) self-reported depression, (c) one or more days lost to work or housework during the past 30-day period, and (d) life satisfaction reports of little or none at all, we placed respondents along a continuum from 0 to 4 on our mental health problems scale, which yielded a Chronbach’s alpha of 0.70. The mean score of respondents on the personal psychological problems scale was 1.6 out of a possible 4, SD = 1.3, with an *N* of 556). We also assessed suicide thoughts with a question taken from the National Survey of Drug Use and Health, “How often during the past 12 months did you think about taking your own life? Answers were graded on a five-point scale from never to very frequently.

To assess positive growth after the loss, we also included the 7 items that had the highest factor loadings from a 12-item set of personal growth questions that formed part of the Hogan Grief Reaction Checklist, HGRC (Hogan et al., 2001). The included items were “I have learned to cope better with life; I feel as though I’m a better person; I have a better outlook on life; I have more compassion for others; I am stronger because of the grief I have experienced; I care more deeply for others; I am a more forgiving person.” These 7 items yielded an alpha coefficient of 0.91 among the 536 respondents that offered useable responses to this abbreviated personal growth scale where responses ranged from a low of 7 to a high of 35, with a mean of 24.0, SD = 7.1).

To assess being stigmatized, two additive scales were summed, one measuring whether any of 11 different groups (such as spouse, ex-spouse, children, siblings, other relatives, coworkers, friends, etc.) acted helpfully or hurtfully to the respondent after the death; the other asking if any of these same 11 different groups had the respondent’s relationship to the group remained the same, was strengthened or weakened after the death. A more detailed discussion of this scale can be found on pages 44 and 45 of Feigelman et al. (2012). The stigmatization scale was completed by 553 respondents, yielding a mean of 3.3, SD = 3.3, with a range from 0 to 16; higher scores meant more unhelpful and/or hurtful responses and more relations being reported as weakening from the diverging groups of intimate associates. The overall stigmatization scale yielded an alpha score of 0.76.

We also had measurements for whether the parent respondent had witnessed the death, or had found the body or not. There were also questions whether the parent had a positive, mixed or negative relationship with their deceased child prior to the death, and whether their suicide deceased child had made prior suicide attempts or not. We also queried whether the bereaved parent had obtained the support of a professional counselor during the past year and their frequency of visits. We collected data on parents’ marital statuses, whether the respondent reported him or herself as happily married or not; whether their child’s death had improved, worsened, or had not effected the quality of their marital relationship. The time since the loss, whether the parent had experienced anger or shock with their child’s death were also investigated.

### Data Analysis

In the first part of this analysis we examined feelings of blameworthiness as an independent variable, first examining its distribution and how it changed with the passage of time after a loss. Then, we examined whether the suicide and drug death bereaved showed any higher levels of feelings of blameworthiness compared to parents whose children died from other accidents or natural death causes. We examined these questions in our complete dataset of all 575 respondents. Then, as we proceeded further, throughout the remainder of this analysis, our focus shifted to the 462 suicide bereaved parents, who were the primary focus of this study. Next, we examined blameworthiness and its associations with seven different important dimensions of mental health and grief problems. In the final parts of this analysis we examined blameworthiness as a dependent variable. We conducted several cross-tabular, correlational and multivariate analyses of blameworthiness as it may be predicted from several other leading competing predictors of grief problems, time since the loss, stigma score, whether the respondent had found the body, whether parents had a happy marriage and whether they had a positive or conflicted relationship with their deceased child prior to the death.

## Results

### Participants

These 575 bereaved parents showed mean years since loss of 5.6 years, ranging from less than a month from after the death till 40 years afterward. Females outnumbered males in our sample by a huge margin, 85–15%. 74% of respondents were between ages 46 and 65, as sample members reported belonging to one of five different age categories. The sample over represented upper-status respondents with a third having yearly incomes of over $90,000 (during the period of 2006), 53% holding managerial or professional occupations and 41% with four years of college or greater education; religious identifications were mixed; the sample was predominately White at 95% and native born at 94%.

### Feelings of Blameworthiness, Contrasts Over Time

Table 1 shows the contrasts between feelings of blameworthiness at the time of the death, contrasted with having these feelings at the time of the research interview. We can see that at the time of the death more than half (55%) of bereaved parent respondents expressed having strong to moderate feelings of blameworthiness; this shrank to only 18% having these same feelings at the time of the research interview.

**TABLE 1:** Comparison of feelings of blameworthiness of participants at time of death vs. at time of research study.

Feelings of blameworthiness	At time of death (%)	At time of interview (%)
Almost completely	207 (36.19)	39 (6.84)
Mostly	107 (18.71)	65 (11.40)
Somewhat	99 (17.31)	163 (28.60)
A little bit	68 (11.89)	125 (21.93)
Almost not at all	91 (15.91)	178 (31.23)
Total sample size	572	570

### Differences in Blameworthiness by Type of Loss

Table 2 shows sharply contrasting differences between suicide and drug death mourners and ordinary accident and natural death causes bereaved parents. Close to three quarters of natural and ordinary accidental death bereaved felt not at all or hardly blameworthy in the death, compared to only about half of the suicide and drug death bereaved. These differences were statistically significant with the chi-square test. We suspected these differences could have been related to differences in time since the loss between these subgroups. To test for this potentially confounding causal influence we regressed feelings of being blameworthy with differences in the subgroup death causes and years since the loss. Both remained statistically significant predictors, suggesting that both factors are significant correlates in accounting for variations in feelings of blameworthiness. These regression results are not displayed in our tables.

**TABLE 2:** Differences in feelings of blameworthiness at time of research interview among different subgroup death cause groups.

Feelings of blameworthiness	Natural death (%)	Suicide (%)	Drug death (%)	Ordinary accident (%)
Almost completely	1 (4.17)	36 (7.81)	1 (2.27)	1 (2.70)
Mostly	1 (4.17)	56 (12.15)	6 (13.64)	2 (5.41)
Somewhat	3 (12.50)	140 (30.37)	13 (29.55)	6 (16.22)
A little bit	9 (37.50)	98 (21.26)	13 (29.55)	5 (13.51)
Almost not at all	10 (41.67)	131 (28.42)	11 (25.00)	23 (62.16)
Total	24	461	44	37

*Chi-square = 29.5386 (12df), *p* = 0.003.*

### Associations Between Feelings of Blameworthiness, Grief Problems, and Mental Health

Next, we examined the interrelations between seven diverging measures of grief difficulties and mental health differences with feelings of blameworthiness (see Table 3). This analysis was conducted exclusively among the 462 suicide bereaved parents. All were significantly associated, showing greater feelings of blameworthiness associated with higher grief difficulties, greater complicated grief, higher PTSD, higher depression scores, more personal psychological problems, and less personal growth. In some instances, high associations indicating correlation coefficients above 0.40 were observed.

**TABLE 3:** Correlations of Seven Grief and Mental Health Measures with Feelings of Blameworthiness (*n* = 462).

Measure	1	2	3	4	5	6	7	8
1. AbbrevGEQ								
2. Complicated Grief	0.7762							
3. PTSD	0.7012	0.6945						
4. Personal Growth	−0.0.3618	−0.5013	−0.2651					
5. Depression	0.5769	0.6270	0.4876	−0.3358				
6. Psych Probs	0.2031	0.2120	0.1178	−0.1150	0.3959			
7. Suicide Thoughts	0.5244	0.5602	0.3593	−0.3969	0.4763	0.2016		
8. Blame Presently	−0.5687	−0.4773	−0.3626	0.2786	−0.3458	−0.1392	−0.3732	

*All were significantly associated (*p* < 0.05).*

### Examining Feelings of Blameworthiness as a Dependent Variable

In Table 4a we display the associations between feelings of blameworthiness and other potentially associated elements that were categorical variables such as marital status. If a respondent indicated witnessing the death (a rarity in this sample) or finding the body (*n* = 154), compared to those not having this experience (*n* = 416) there were no significantly greater feelings of blameworthiness (*p* = 0.22). Marital status differences and whether the child had made one or more previous attempts prior to the suicide were not associated with differences in feelings of blameworthiness. A near significant association was noted between feelings of blameworthiness and whether the respondent reported the child’s death had improved their marital relationship, compared to those reporting worsened relationships (*p* = 0.09). Table 4a also shows that feelings of blameworthiness were higher when the bereaved parent had a mixed, or negative relationship with their child, compared to a positive relationship (*p* = 0.06).

**TABLE 4A:** Associations between feelings of blameworthiness and categorical variables. *N* = 462.

Variables	Almost completely (%)	Mostly (%)	Somewhat (%)	A little (%)	Almost none (%)
Witnessing death or finding body					
No	22 (7.07)	38 (12.22)	93 (29.90)	60 (19.29)	98 (31.51)
Yes	14 (9.33)	18 (12.00)	47 (31.33)	38 (25.33)	33 (22.00)
Total	36 (7.81)	56 (12.15)	140 (30.37)	98 (21.26)	131 (31.23)
Child made prior suicide attempts					
No	24 (7.92)	33 (10.89)	85 (28.05)	74 (24.42)	87 (28.71)
Yes, one	5 (6.17)	11 (13.58)	23 (28.40)	17 (20.99)	25 (30.86)
Yes, two	7 (9.33)	11 (14.67)	31 (41.33)	7 (9.33)	19 (25.33)
Total	36 (7.84)	55 (11.98)	139 (30.28)	98 (21.35)	131 (28.54)
Current marital status					
Married	21 (6.77)	33 (10.65)	96 (30.97)	71 (22.90)	89 (28.71)
Divorced	10 (9.80)	16 (15.69)	29 (28.43)	17 (16.67)	30 (29.41)
Separated	0 (0.00)	2 (16.67)	6 (50.00)	1 (8.33)	3 (25.00)
Never	2 (33.33)	1 (16.67)	1 (16.67)	0 (0.00)	2 (33.33)
Widowed	3 (10.34)	4 (13.79)	4 (13.79)	8 (27.59)	7 (24.14)
Total	36 (7.84)	56 (12.20)	139 (30.28)	97 (21.13)	131 (28.54)
Marriage relationship changed after the death					
Weaker	12 (15.19)	11 (13.92)	25 (31.65)	16 (20.25)	15 (18.99)
Same	4 (6.56)	9 (14.75)	17 (27.87)	15 (24.59)	16 (26.23)
Stronger	9 (4.57)	21 (10.66)	63 (31.98)	41 (20.81)	63 (31.98)
Total	25 (7.42)	41 (12.16)	105 (31.16)	72 (21.36)	94 (27.89)
Relationship with deceased prior to the death					
Pos Rel	26 (6.93)	41 (10.93)	110 (29.33)	82 (21.87)	116 (30.93)
Mixed/Neg	10 (11.90)	14 (16.67)	29 (34.52)	16 (19.05)	15 (17.86)
Total	36 (7.84)	55 (11.98)	139 (30.28)	98 (21.35)	131 (28.54)

*Chi-square for Witness/find and Blame association = 5.6921 (4 df), pr = 0.223; Chi square for Priorattempt and Blame association = 11.885, (8 df), pr = 0.156; Chi-square for Marital Status and Blame association = 16.0332 (16 df), pr = 0.451; Chi square for Relations Changed wSpouse and Blame = 13.6710 (8 df) pr = 0.091; Chi-square for Relsw/deceased Before Death and Blame = 9.0227 (4 df), pr = 0.061.*

**TABLE 4B:** Correlates of feelings of blameworthiness. *N* = 462.

Factors	1	2	3	4	5	6	7
1. Saw Prof Counsel							
2. Stigma Score	0.2146*						
3. Happily married	−0.1368*	−0.2843*					
4. Shock	0.0497	−0.0537	0.0247				
5. Anger	−0.0286	−0.0735	−0.0201	0.1643*			
6. Years since loss	−0.2585*	−0.0492	0.0361	0.0401	0.0419		
7. Blame presently	−0.1156*	−0.2015*	0.2995*	0.0854	−0.0970*	0.1014*	

*^∗^*p* < 0.05.*

This examination of categorical variables and blameworthiness must be done for examining marital status differences but could be substituted with correlational tests where only two categories were available, with one variable being coded as 0 and the other as 1, or in the case of positive, vs. negative or unchanged marital adjustments where the numeric categories could be −1, 0, and +1. We computed correlation coefficients for all variables in Table 4a with the exception of marital status. The results were as follows: finding the body, −0.06, *p* = 0.16; prior suicide attempts, −0.07, *p* = 0.12; whether the parent’s relationship strengthened, weakened or remained the same, 0.16, *p* = 0.002; the relationship with their child, before the death, whether positive or mixed/negative, −0.139, *p* = 0.002. These correlation coefficients yielded potential associations that the bivariate cross-tabs did not show. However, it will be in a multiple regression analysis comparison where these bivariate associations can be more meaningfully investigated, in the presence of potentially competing explanations.

Table 4b presents a correlation matrix showing the relationships between feelings of blameworthiness and six different other potential associated factors: whether the respondent had sought professional counseling services during the past year and their frequency of use, stigmatization scale scores, assessments of the happiness of their marriage as measured on a ten-point scale, the degree on a five-point scale that they felt shocked by the death when it occurred; the degree on a five-point scale they felt anger from the death when it occurred and time since loss, ranging from less than a month to 40 years afterward. In Table 4b almost all of these hypothesized associated variables were significantly associated with feelings of blameworthiness with the exception of feeling shocked at the time of the death or at the time of the research interview. Only two associations here seemed sufficiently robust to be important: stigma scale scores with a correlation coefficient of 0.20, and marital happiness with a correlation of nearly 0.30.

### Multiple Regression Analysis of Feelings of Blameworthiness and Potential Confounding Variables

We anticipated that there might be some overlapping influence between all these potential predictors of feelings of blameworthiness. To eliminate these confounding causal influences, we conducted a multiple regression analysis of all significant correlates shown in Tables 4a,b. This is displayed in Table 5a. The multiple regression analysis showed only three predictors significantly associated with feelings of blameworthiness: whether or not the respondent had a positive relationship with their child prior to the death, their total stigmatization scale score and whether or not they reported themselves as happily married. All the remaining other correlates in this regression model were redundant: whether they had currently sought help from a mental health professional, whether they were shocked at the death, time since the loss, whether spousal relations had improved or worsened since the death and the number of their child’s prior suicide attempts. Although some of these factors had yielded significant associations with feelings of blameworthiness in univariate correlation analyses, in the multiple regression, these variables were found to be non-essential. The three significant variables – stigmatization scale score, whether the parents reported having a happy marriage and a parent’s relationship to their child prior to the death – explained 16% of the differences in blameworthy scores.

**TABLE 5:** Multiple regression – predictors associated with feelings of blameworthiness among married couples only.

(A) Predictor	Coefficient	Beta weight	*p*-value
Prior Relsw/decease	**−**0.370	**−**0.117	0.044**
Saw Prof Counsel	0.010	0.012	0.837
Stigma Score	**−**0.063	**−**0.177	0.004**
Happily married	0.158	0.208	0.004**
Shock	0.093	0.112	0.064
Years since death	0.028	0.103	0.082
Witness/find	**−**0.126	**−**0.047	0.404
Spouse Rels Chang	0.035	0.022	0.740
Prior attempt	**−**0.076	**−**0.049	0.436

**(B) Predictor**	**Coefficient**	**Beta weight**	***p*-value**

Prio Relsw/decease	**−**0.474	**−**0.146	0.002**
Saw Prof Counsel	**−**0.037	**−**0.046	0.333
Stigma Score	**−**0.074	**−**0.198	0.000**
Years since death	0.012	0.045	0.333
Witness/find	**−**0.139	**−**0.052	0.251
Shock	0.071	0.083	0.069

*No. of obs. = 279; *F* (9,269) = 5.58; Prob. > *F* = 0.0001; *R*^2^ = 0.1573. Multiple regression – predictors associated with feelings of blameworthiness among all suicide bereaved parents. No. of obs. = 449; *F* (6,442) = 7.12; Prob. > *F* = 0.0001; *R*^2^ = 0.09. ^∗∗^*p* < 0.05.*

We were somewhat surprised that time since loss, which has been shown in so many bereavement studies to be an enduring and significant correlate of grief problems could be washed out so easily in the multiple regression equation. It should be noted that the first multiple regression equation was based on only 279 cases, only those currently married. Our complete sample of suicide bereaved consisted of 462 respondents. In a second multiple regression, we re-ran all variables except those that made the analysis exclusively of the currently married suicide bereaved. In Table 5b we show the same regression without the two variables that narrowed the scope of the analysis to be exclusively of married couples, omitting the variables marital happiness and whether the marital adjustment had been strengthened or worsened since the death. This added 170 more cases of unmarried, divorced and/or widowed suicide bereaved parents into the analysis. Here, again the results were remarkably similar with the results shown in the Table 5a with two significant predictors, whether the respondent had a positive or mixed or negative relationship prior to the death and the total stigmatization score.

## Discussion

Our findings showed feelings of blameworthiness closely associated with grief difficulties, complicated grief, PTSD, depression and other psychological problems. These findings are probably not all that surprising. The especially high correlations between feeling of blame, grief difficulties and complicated grief derives from many similar items about feeling blame and guilt being embedded within these scales; it is therefore no wonder that there are such high levels of association. Probably the most useful take-away point from these results is that feelings of blameworthiness can serve a good short hand indicator of the presence of grieving difficulties.

Another finding meriting comment was our results showing feelings of blameworthiness surging when the bereaved had a mixed, or negative relationship with their child, compared to a positive relationship. Almost twice as many parents felt not at all blameworthy in their child’s death when they had reported a positive relationship with their child (31%), compared to only 18% who reported negative or mixed relations with their child. We can see that guilt may be generated after the death from not sharing the normative expectations of having a positive relationship with one’s child.

Perhaps our most noteworthy and surprising finding were in the multiple regression results showing time since loss being eclipsed by the three variable combination of stigmatization scores, levels of marital happiness and the relationship of the parent with the deceased child prior to the death. We suspected that the stigmatization scores hold the key to understanding why time since the death was easily overshadowed by this three-variable combination. Most of the negative or unhelpful comments that bereaved parents had heard following the deaths of their children were expressed by ex-spouses, parents, parent-in laws and siblings. These close family groups much more frequently reported offering unhelpful and hurtful responses rather than other groups such as, friends, co-workers, neighbors and acquaintances. Many of the comments made were critical or unsupportive comments like: “how come you didn’t call him that last night?^1^” (the night of the suicide or the fatal overdose); “Couldn’t you have gotten him into a better a treatment program/or have taken him to a better therapist.” Much of the advice might have been “I wouldn’t have done that with my child.” At support group meetings, many bereaved shared these upsetting, guilt-inducing, second guessing comments that they found highly distressing. Many bereaved longed for support and empathic responses from close family members, and sometimes stood in stark disbelief that a so-called beloved close relative could say such hurtful things to them when they desperately sought approval, support and validation from close family members.

Suicide bereaved parents themselves are inclined to blame themselves in the deaths of their children, no matter what. They cannot help themselves from wondering, what if I had done this, called or visited at this time, given more aid, done this or that, just before the death, if this would have meant the difference in saving the life of their child. This wonderment, guilt, and endless speculation is especially keen among mothers. Rynearson (2011) claims that mothers are socialized to feel completely responsible for the welfare and well-being of their children. If one dies or falls sick they feel they have failed abysmally from performing their time honored role as mother, keeping their child alive at all costs. Thus, the tiniest lack of support or compassion expressed by intimate family members goes a long way toward engendering greater guilt and blame in the fragile mind of a suicide or drug death bereaved parent.

Our findings showing marital unhappiness associated with feelings of blameworthy are not at all unexpected. While many suspect that the traumatic death of a child impairs their marital adjustments our results did not confirm this speculation (Feigelman et al., 2012, Chapter 13). More than twice as many of the married respondents in our sample reported having closer relations with their partner following their child’s suicide or drug death, as those reporting more impaired relationships (see Table 4a). In a minority of families, the child’s suicide or drug death leads to a rupture in their relationship. It is likely for many, a gulf may have existed in these couple’s lives prior to the deaths. When the couple experienced harmony and support with each other, in these cases, their children’s deaths usually brought them closer together, with the additional result of lessening feelings of guilt and blame.

These findings suggest that receiving immediate and unconditional support from close family members will overshadow time since loss in determining positive bereavement outcomes. When close family members continue to second-guess and withhold support, long after the occurrence of the death, feelings of blameworthiness and guilt will emerge or persist in the mind of the bereaved, leading them to spiral downward in their adaptations. Further research confirming these results will be necessary for determining the appropriate kinds of help and services essential for these traumatically bereaved.

We should acknowledge the limitations of this study. Most importantly it was based on a convenience sample of volunteers with a high oversampling of females who remain dominant in the support groups where most respondents were selected from. It should be noted there is bound to be some over-representation of women even in socially representative samples of the suicide bereaved by virtue of the sharp disparity of men taking their lives over women, with a 3 to 1 ratio of differences (Feigelman et al., 2018). We should also note that our respondents over-represented higher status, whites which could yield diverging results from samples collected with a greater representation of minorities. Though this data was originally collected over ten years previously, we doubt that these results would vary much from what might be found in a more contemporaneous sample.

Had we foreseen this research project emerging beforehand we would have been better off having several questions devoted to measuring feelings of blameworthiness in the death of a loved one, rather than a single question. Ideally, it also would have been more advantageous to study the feelings of blameworthiness longitudinally, especially over a period of three to five years after the death to examine their transformations over time. These acknowledgments and the many fruitful nuggets of new information obtained here should inspire future researchers to further investigate this potentially rich realm of blameworthy feelings correlating ever so closely with complicated grief, grief problems and a bereaved person’s mental health. Some new research by Levi-Belz and Gilo (2020) suggests that self-forgiveness represents an important component in transitioning from high self-blame after a loved one’s suicide into adopting lower, healthier levels of these feelings, which demands much further investigation and study.

## Data Availability Statement

The datasets generated for this study are available on request to the corresponding author.

## Ethics Statement

The studies involving human participants were reviewed and approved by the IRB Nassau Community College. The patients/participants provided their written informed consent to participate in this study.

## Author Contributions

Both authors contributed to the development and writing of this report.

## Conflict of Interest

The authors declare that the research was conducted in the absence of any commercial or financial relationships that could be construed as a potential conflict of interest.

## Footnotes

1 These comments were shared at survivor of suicide support group meetings attended by the first author from April 2017 to December 2019. Comments were made by various family members related to the support group members who were grieving their suicide losses.
